# Toddler temperament and prenatal exposure to lead and maternal depression

**DOI:** 10.1186/s12940-016-0147-7

**Published:** 2016-06-16

**Authors:** Annemarie Stroustrup, Hsiao-Hsien Hsu, Katherine Svensson, Lourdes Schnaas, Alejandra Cantoral, Maritsa Solano González, Mariana Torres-Calapiz, Chitra Amarasiriwardena, David C. Bellinger, Brent A. Coull, Martha M. Téllez-Rojo, Robert O. Wright, Rosalind J. Wright

**Affiliations:** Department of Pediatrics, Icahn School of Medicine at Mount Sinai, New York, NY USA; Department of Preventive Medicine, Icahn School of Medicine at Mount Sinai, New York, NY USA; Department of Developmental Neurobiology, National Institute of Perinatology, Mexico City, Mexico; Center for Nutrition and Health Research, National Institute of Public Health, Morelos, Mexico; Departments of Neurology and Psychiatry, Boston Children’s Hospital, Harvard Medical School, Boston, MA USA; Department of Environmental Health, Harvard School of Public Health, Boston, MA USA; Department of Biostatistics, Harvard School of Public Health, Boston, MA USA; Division of Newborn Medicine, Department of Pediatrics, Icahn School of Medicine at Mount Sinai, One Gustave L. Levy Place, Box 1508, New York, NY 10029 USA

**Keywords:** Temperament, Prenatal exposure, Lead, Depression in pregnancy, Neurobehavioral outcomes

## Abstract

**Background:**

Temperament is a psychological construct that reflects both personality and an infant’s reaction to social stimuli. It can be assessed early in life and is stable over time Temperament predicts many later life behaviors and illnesses, including impulsivity, emotional regulation and obesity. Early life exposure to neurotoxicants often results in developmental deficits in attention, social function, and IQ, but environmental predictors of infant temperament are largely unknown. We propose that prenatal exposure to both chemical and non-chemical environmental toxicants impacts the development of temperament, which can itself be used as a marker of risk for maladaptive neurobehavior in later life.

In this study, we assessed associations among prenatal and early life exposure to lead, mercury, poverty, maternal depression and toddler temperament.

**Methods:**

A prospective cohort of women living in the Mexico City area were followed longitudinally beginning in the second trimester of pregnancy. Prenatal exposure to lead (blood, bone), mercury, and maternal depression were assessed repeatedly and the Toddler Temperament Scale (TTS) was completed when the child was 24 months old. The association between each measure of prenatal exposure and performance on individual TTS subscales was evaluated by multivariable linear regression. Latent profile analysis was used to classify subjects by TTS performance. Multinomial regression models were used to estimate the prospective association between prenatal exposures and TTS performance.

**Results:**

500 mother-child pairs completed the TTS and had complete data on exposures and covariates. Three latent profiles were identified and categorized as predominantly difficult, intermediate, or easy temperament. Prenatal exposure to maternal depression predicted increasing probability of difficult toddler temperament. Maternal bone lead, a marker of cumulative exposure, also predicted difficult temperament. Prenatal lead exposure modified this association, suggesting that joint exposure in pregnancy to both was most toxic.

**Conclusions:**

Maternal depression predicts difficult temperament and concurrent prenatal exposure to maternal depression and lead predicts a more difficult temperament phenotype in 2 year olds. The role of temperament as an intermediate variable in the path from prenatal exposures to neurobehavioral deficits and other health effects deserves further study.

## Background

Temperament reflects the manner in which an individual interacts with and responds to social and emotional environmental cues. Distinct temperament traits are identifiable in infancy and are relatively stable over time [1, 2]. Temperament is a biologically based trait [3] and is associated with later child behavior, both normal and pathological [4–7]. Early childhood temperament can be seen as an important risk factor for behavioral problems that are not expressed until later in life as well as for diseases such as obesity [8–14]. While the full range of risk factors that predict maladaptive temperament are not well-understood, it is believed that temperament is established during perinatal life through both genetic and environmental factors [13–16]. As such, *in utero* exposure to neurotoxicants might impact infant temperament, and maladaptive temperament may be an early life intermediate phenotype for behavioral disorders brought on by environmental toxicants.

Although prenatal exposure to elemental metals including lead and mercury is known to be detrimental to cognition and specific behavioral domains in childhood [17–23], the relationship between *in utero* metal exposure and temperament has not been studied previously. Social stressors such as maternal mental state and stress level – which we refer to as “non-chemical toxicants” [24] – can also have a significant clinical impact on the developing fetus [25–30]. Women who experience toxic stress, anxiety, or depression while pregnant have children at elevated risk for certain physical, cognitive, and behavioral difficulties [28–32]. To our knowledge, no study has attempted to address whether concurrent prenatal exposure to chemical and non-chemical stressors interact to predict maladaptive temperament phenotypes.

While gene-environment interactions have received much attention, the concurrent exposure to multiple toxins may also multiplicatively increase effects seen in single exposure models [33–42], and studies of prenatal exposure should evaluate both chemical and non-chemical exposures to reflect real-life exposure scenarios. In this study, we hypothesized that prenatal co-exposure to chemical and non-chemical neurotoxicants is associated with difficult infant temperament. The study was nested in a large prospective environmental health cohort study designed to investigate the impact of concurrent prenatal exposure to lead, mercury, maternal depression, socioeconomic status, and maternal nutrition on multiple long-term neurobehavioral outcomes.

## Methods

### Participant identification and enrollment

Between 2007 and 2011, healthy pregnant women in Mexico City were recruited through the Mexican Social Security System to participate in the PROGRESS (Programming Research in Obesity, GRowth, Environment, and Social Stress) birth cohort [43]. Informed consent to participate in the study was obtained from participants. Nine hundred forty-eight women were enrolled prior to 20 weeks of pregnancy and delivered a live infant. Of these, 760 children were evaluated for neurodevelopment in at least one visit at 6, 12, 18, or 24-months of age. Of the 549 completing the 24-month visit, 500 mothers responded to the Toddler Temperament Scale (TTS), the primary outcome in these analyses. Research ethics committees of the participating institutions approved the study (the Comité de Investigación, Comité de Bioseguridad, and the Comité de Ética en la Investigación of the National Institute of Public Health, Mexico, the Partners Human Research Committee at Brigham and Women’s Hospital, the Office of Human Research Administration at the Harvard School of Public Health, and the Program for the Protection of Human Subjects at the Icahn School of Medicine at Mount Sinai).

### Participant data collection

Questionnaires were administered to collect sociodemographic information including maternal age, parity, education level, and socioeconomic status (SES). Thirteen variables derived from questionnaire results were used to classify study participant families into six levels based on the SES index created by the Asociación Mexicana de Agencias de Investigación de Mercados y Opinión Pública [44]. These levels were then collapsed into low, medium, and high socioeconomic status.

### Lead measurements

Maternal lead exposure was assessed by inductively coupled plasma-mass spectrometry (Agilent 8800, Santa Clara, CA) of maternal blood during the second trimester and in bone by maternal in vivo K-shell X-ray fluorescence (K-XRF) one month postpartum (ABIOMED, Danvers, MA, USA) [45, 46]. Blood lead measurement quality control (QC) and quality assurance procedures used were: included analyses of procedural blanks, duplicates, spiked samples, national institute of standard reference material (NIST SRM) 955 (Lead in Blood); NIST SRM 1643e (trace elements in water) and blood samples from the inter-laboratory study program INSPQ/Laboratoire de Toxicologie, Quebec to monitor the accuracy and recovery rates of the procedure for each analytic batch. Lab recovery rates for QC standards and spiked samples with this method were 9- 110 % and precision (given as % RSD) was <5 %. The limit of detection for this procedure was 0.2 ng ml-1.

Our K-XRF protocol measures bone lead at the mid-shaft tibia (cortical bone) and the patella (trabecular bone) [47] to provide a representation of cumulative fetal lead exposure through gestation [45, 48]. K-XRF produces negative values when the bone lead content is below the detection limit of the instrument. As imputation to replace negative values in this context has not been shown to be beneficial [49], we included negative values as reported by K-XRF in our analyses.

### Mercury measurements

Maternal mercury exposure was evaluated by measuring mercury deposition in maternal toenails collected during pregnancy. Toenails from all ten toes were collected and pre-cleaned by sonicating for 15 min in approximately 10 mL of 1 % Triton X-100 solution to remove extraneous contaminants. Samples were then rinsed with distilled deionized water and dried at 60 °C for 24 h in a drying oven. Mercury level was assessed using the Direct Mercury Analyzer 80 (Milestone Inc., Monroe, CT) using previously published methods [50]. Samples were analyzed using an aqueous calibration standards and verifyied using different weights of certified reference material GBW 07601 (human hair; Institute of Geophysical and Geochemical Exploration, China). Mercury recovery was 90–110 %, with greater than 90 % precision. The detection limit for samples varied according to sample weight. Sample weight varied from 0.006 g to 0.0.083 g, and the detection limit varied from 0.006 μg/g to 0.08 μg/g (mean = 0.019 μg/g).

### Measurements of child development

All children were assessed for motor, language and cognitive development using the Bayley Scales of Infant and Toddler Development, 3rd edition (BSID) at 24 months of age [51]. Child temperament was assessed at 24 months of age using the TTS [2], which comprises a number of age-appropriate questions. These questions evaluate the nine temperamental characteristics first described by Thomas et al. [52], and expands on earlier work by Carey measuring temperament in infants [53, 54]. Caregivers are presented with a statement describing a certain behavior and asked to rate how often their child behaves in that way on a 6-point scale. The results are coded so that higher scores indicate more difficult temperament. Although the TTS is designed to be completed independently by parents, in the current study trained psychologists verbally administered the TTS to the enrolled mother at the 24-month study visit. In 9 cases where the mother was not present at the 24-month visit, the TTS questionnaire was mailed to the home for independent completion by the mother.

### Measurement of maternal depression risk

The Edinburgh Postnatal Depression Scale (EPDS) was administered to mothers in the second trimester. The EPDS is a ten-item self-report scale designed to identify women experiencing depressive symptoms [55]. It is validated for repeated use during and both immediately and long after pregnancy [56, 57]. For these analyses, the second trimester EPDS result was used as the prenatal depression risk score. If the EPDS was not administered during the second trimester, the third trimester result was used as the prenatal depression risk score. The 24-month EPDS score was obtained at the same study visit as the TTS. Depression risk was evaluated both as a continuous and as a categorical variable dichotomized at an EPDS score of 13, the recommended cut point [55, 58, 59].

Although the focus of this study was to prospectively evaluate the impact of prenatal exposures including maternal depression, we also evaluated the EPDS score obtained cross-sectionally at child age 24-months in analyses, as the caregiver’s mental state at the time of TTS administration has been associated with the assessment of the child’s temperament [60, 61]. As in other studies where depressive symptoms years after childbirth were associated with perinatal depression [62, 63], the categorical variable for prenatal maternal depression risk was associated with depression risk at 24 months (*p* < 0.001), and the continuous variables were correlated (*ρ* = 0.66) in our cohort. To address the collinearity induced by including both antenatal and 24-month post-partum depression in the same model, we included variables for both the average of the two scores and the difference between scores in all analyses using the EPDS continuous variable. This transformation from the original correlated measurements to the sum and difference is the basis for traditional MANOVA analyses for multivariate outcomes [64]. The adjusted representation of the prenatal maternal depression score is referred to as “adjusted EPDS” or “adjusted prenatal depression” throughout this manuscript.

### Statistical approach

#### Linear regression modeling

We examined univariate descriptive statistics, bivariate associations, and multivariable linear regression of each of the 9 TTS subscales using multiple measures of prenatal lead exposure, prenatal mercury exposure, maternal education, SES index, maternal ferritin during pregnancy (as a marker of nutritional status), and prenatal maternal depression score. Interaction terms for different exposures were also examined. Initial models included maternal ferritin and education, but these were dropped from subsequent models as they were insignificant (ferritin) or strongly correlated with the SES index (maternal education). These analyses were conducted using SAS 9.4 (Research Triangle Institute, Cary, NC).

In order to reduce the dimensionality of the overall nine outcome scale into a smaller set of discrete categories, we also used latent profile analysis (LPA) to evaluate associations between multiple prenatal exposures and temperament phenotype.

#### Latent profile analysis

Latent profile analysis (LPA) is a probabilistic, model-based variant of traditional nonhierarchical cluster analysis [65]. We used it to objectively classify children into discrete data-driven temperament profiles. LPA assumes that the population consists of a number of unobserved subgroups that are referred to as latent profiles. Individuals within a given latent profile will show similarities in their TTS subscales scores. For example, children in the same latent profile may demonstrate a high score on subscales for mood, activity and approach, low scores on rhythmicity and adaptability, and intermediate scores on the remaining subscales. Sharing a latent profile does not require consistent high or low scoring across all subscales. Rather, it identifies groups of subjects who have a similar performance pattern globally. Because the TTS assesses multiple behavioral domains that contribute to complex temperament phenotypes, we used LPA to reduce the temperament subscale data into discrete categories. We used Bayesian information criteria (BIC) whereby the smallest BIC value indicates the best fit as well as minimizes cross-classification probabilities. BIC has been shown to identify the appropriate number of profiles in finite mixture models [66] and penalizes the models for a number of parameters that may indicate model over-fit. LPA was implemented on the nine TTS subscales by using a normal mixture model as a model-based clustering technique, as fitted via an expectation–maximization algorithm in the mclust 4.3 R software package [67].

#### Multinomial logistic regression

**Fig. 1:**
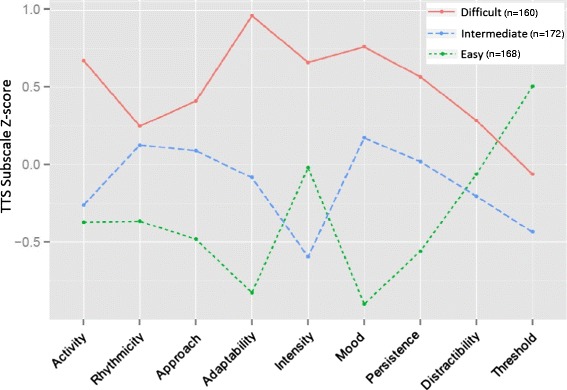
Distribution of TTS Subscale Performance Z-scores by Latent Profiles. Although each profile demonstrates a mix of “easy”, “intermediate”, and “difficult” temperamental traits on varying subscales, profile 1 children generally demonstrated a more difficult temperament, profile 2 generally demonstrated more intermediate temperamental traits, and profile 3 children generally demonstrated an easy temperament

Multinomial Logistic Regression (MLR) is an extension of logistic regression, which analyzes dichotomous variables. In multinomial logistic regression analysis, three or more dependent nominal variables (i.e. not clearly in defined order) are regressed choosing one as the referent group. Our LPA defined three groups (Fig. 1), two of which we could clearly categorize as easy vs difficult, with the third group being intermediate. Upon selection of a final latent class fit, we used MLR analysis to evaluate concordance between identified TTS profiles and performance on the BSID, to confirm that the identified profiles represented behaviorally consistent groupings. Based on prior noted associations between temperament and language performance [52, 68–70], we expected children with the more difficult temperament profile to be associated with worse performance on BSID measures of language. We then regressed our prenatal exposures of interest with the LPA categories [69]. Specifically, MLR was used to evaluate associations between TTS profiles and measures of prenatal lead exposure, Hg exposure, SES, and maternal depression using “easy” as the referent group. Blood lead levels were natural log transformed. Probabilistic models were built to evaluate the association between temperament profile and concurrent exposure to varying levels of prenatal factors that were found to be significant in single exposure models. LPA modeling and graphics were completed using R (R Core Team, Vienna, Austria, 2014) [71].

## Results

Table 1 summarizes the demographic data on the 500 mother-infant pairs included in these analyses. Our study cohort, those who completed the TTS, were significantly more likely to be of low SES and less likely to be of high SES than PROGRESS participants who did not complete the TTS (*p* = 0.03). There were no other significant demographic differences between the groups. Table 2 presents associations between chemical and non-chemical prenatal exposures and scores on individual TTS subscales. Adjusted prenatal depression risk score, SES classification, maternal tibia lead K-XRF, and prenatal maternal toenail mercury levels were associated with TTS subscale performance in single exposure models. Interaction models including adjusted prenatal depression risk score and either second trimester maternal blood lead, maternal tibia lead K-XRF, or second trimester maternal toenail mercury were also significantly associated with certain TTS subscales

**Table 1:** Characteristics of the study cohort compared to the parent PROGRESS cohort and the general Mexican population

Participant Characteristics	Mexican population [44]	Selected study cohort (*n* = 500)	PROGRESS families not in the selected cohort (*n* = 260)
Continuous variables			
Maternal age (years)		26.9 ± 5.5	27.3 ± 5.3
Maternal 2nd trimester blood Pb, (median (IQR), ug/dL)		2.8 (2.7)	2.8 (2.4)
Maternal postpartum tibia Pb (mean ± SD, ug/g bone mineral)		2.6 ± 8.6	2.7 ± 8.3
Maternal postpartum patella Pb (mean ± SD, ug/g bone mineral)		4.9 ± 8.9	4.3 ± 8.0
Maternal EPDS score, 2nd or 3rd trimester (mean ± SD)		8.3 ± 5.7	8.9 ± 6.0
Categorical variables			
Maternal education			
More than high school		117 (23.4 %)	60 (23.1 %)
High school		171 (34.2 %)	104 (40.0 %)
Less than high school		212 (42.4 %)	96 (36.9 %)
Socioeconomic index			
High	21.2 %	46 (9.2 %)	33 (12.7 %)
Middle	53.7 %	177 (35.4 %)	105 (40.4 %)
Low	25 %	277 (55.4 %)	122 (46.9 %)

**Table 2:** Associations Between Prenatal Exposures and TTS Subscales [β (*p*-value)] by Linear Regression Modeling

	Activity	Rhythmicity	Approach	Adaptability	Intensity	Mood	Persistence	Distractibility	Threshold
1. SES	−0.014 (0.54)	−0.081 (0.005)*	−0.027 (0.47)	−0.017 (0.56)	−0.026 (0.28)	−0.040 (0.12)	0.013 (0.55)	0.034 (0.19)	0.067 (0.030)*
2. EPDS	0.021 (0.0003)*	0.029 (<0.0001)*	0.020 (0.037)*	0.031 (<0.0001)*	0.005 (0.46)	0.032 (<0.0001)*	0.018 (0.001)*	0.008 (0.24)	−0.020 (0.015)*
3. Blood lead	−0.009 (0.38)	0.016 (0.21)	−0.012 (0.46)	0.018 (0.17)	−0.005 (0.63)	0.005 (0.67)	0.001 (0.91)	−0.002 (0.84)	−0.009 (0.53)
4. Blood lead x EPDS	<0.001 (0.98)	−0.005 (0.11)	−0.004 (0.37)	−0.003 (0.46)	0.006 (0.05)*	0.001 (0.74)	0.004 (0.11)	0.004 (0.20)	0.005 (0.14)
5. Tibia lead	−0.002 (0.56)	0.004 (0.32)	−0.001 (0.79)	−0.0008 (0.86)	−0.002 (0.67)	0.0008 (0.84)	−0.003 (0.42)	−0.006 (0.11)	−0.011 (0.017)*
6. Tibia lead x EPDS	<0.0001 (0.99)	−0.003 (0.01)*	−0.002 (0.22)	−0.001 (0.31)	−0.0003 (0.73)	−0.0008 (0.37)	0.0007 (0.38)	−0.0009 (0.37)	−0.0001 (0.93)
7. Mercury	0.12 (0.49)	0.28 (0.21)	−0.25 (0.40)	0.16 (0.50)	0.35 (0.07)	0.10 (0.61)	−0.13 (0.45)	0.30 (0.16)	0.090 (0.71)
8. Mercury x EPDS	0.088 (0.04)*	0.053 (0.33)	0.002 (0.97)	0.13 (0.03)*	0.022 (0.64)	0.071 (0.15)	0.028 (0.51)	0.031 (0.55)	0.091 (0.13)

Maternal blood lead and toenail mercury were measured in the second trimester of pregnancy. Tibia lead was measured by K-XRF one month post-partum

Models 2–8 were adjusted for SES

Models 4, 6, and 8 evaluated the interaction between exposures of interest

*N* = 500

*p ≤ 0.05

Neither maternal patella K-XRF, nor interaction models using patella lead measurement and adjusted prenatal depression risk were associated with TTS subscale scores. Similarly, interaction models between prenatal mercury level and any measure of prenatal lead exposure were not significantly associated with TTS subscale performance (results not shown).

**Fig. 2:**
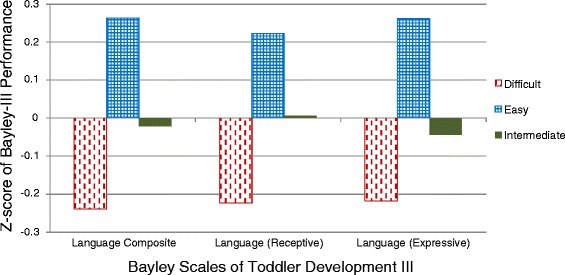
Performance on the Language Scales of the BSID by Temperament Profile. As expected based on prior studies of temperament and language ability [68, 70, 72], children in the easy temperament profile performed well while children in the difficult temperament profile performed poorly

Our LPA demonstrated three discrete profiles (Fig. 1). Children fell into three categories of temperament, two of which could be characterized as difficult (more intense, less regulated, etc.), versus easy-going (less intense, more regulated, etc.). The third category appears to be intermediate, with performance in the moderate range for most subscales. To confirm that our LPA profiles represent neurobehaviorally meaningful groupings, we evaluated the relationship between our study’s TTS profiles and language performance on the BSID. Figure 2 shows how children in each profile performed on the BSID language elements. Consistent with studies in other populations [68, 70, 72], more difficult toddler temperament was significantly associated with worse performance on tests of language ability in our cohort.

We next tested whether the probability of a subject demonstrating a given TTS profile varied in relation to prenatal exposures. Results of multinomial logistic regression analyses of the relationship between prenatal exposures and TTS latent profile are summarized in Table 3. Greater prenatal exposure to maternal depression estimated by the adjusted prenatal EPDS, and to lead estimated either by maternal blood lead level or K-XRF of the maternal tibia, was associated with significantly increased odds of having a difficult temperament. Prenatal SES, maternal patella lead measurement, and antenatal maternal toenail mercury level were not significantly associated with the temperament classification.

**Table 3:** Associations Between Prenatal Exposures and TTS Profile by Multinomial Logistic Regression Modeling

	EPDS^a^			SES			Blood lead^b^			Tibia lead^c^			Patella lead^c^			Mercury^c^		
Temperament	OR	95 % CI		OR	95 % CI		OR	95 % CI		OR	95 % CI		OR	95 % CI		OR	95 % CI	
Easy	Ref	--	--	Ref	--	--	Ref	--	--	Ref	--	--	Ref	--	--	Ref	--	--
Intermediate	1.23	0.78	1.93	0.71	0.26	1.9	0.88	0.59	1.3	1.25	0.95	1.65	0.99	0.96	1.02	0.82	0.11	6.15
Difficult	2.53	1.69	3.77	1.38	0.46	4.15	1.52	1.03	2.26	1.32	1.01	1.73	1.01	0.98	1.04	1.89	0.29	12.28

^a^Odds ratio (OR) and 95 % CI corresponding to IQR change in adjusted EPDS score (IQR = 7.3)

^b^OR and 95 % CI corresponding to 1 unit change of ln(maternal blood lead ug/dl), which corresponds to a 2.7 fold increase in second trimester maternal blood lead level

^c^OR and 95 % CI corresponding to per 1 unit change per 10 ug/g change in metal measurement

**Fig. 3:**
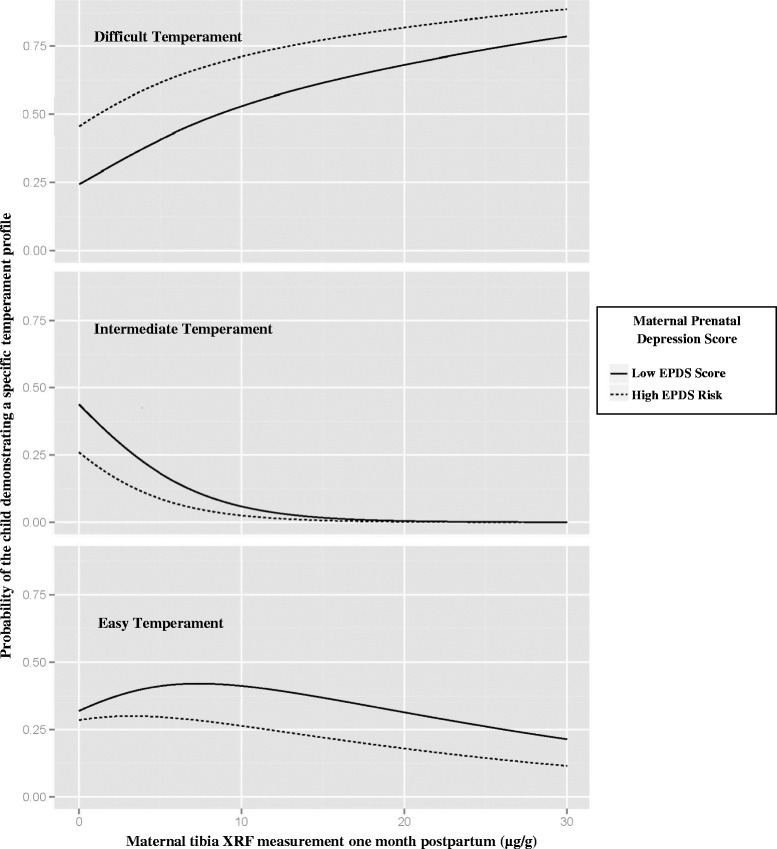
Relationship of Prenatal Exposure to Maternal Depression, Lead, and Toddler Temperament. The probability of demonstrating each of the three temperament profiles (y-axis for each panel) was impacted by both increasing prenatal lead exposure (maternal tibia XRF; x-axis) and adjusted prenatal EPDS score (dotted versus solid lines; dichotomized at 13)

Finally, we addressed concurrent prenatal exposure to factors significant in single predictor models. Figure 3 demonstrates the probability of demonstrating each of the three temperament profiles (y-axis) stratified by adjusted prenatal EPDS score dichotomized at 13, and illustrates the joint impact of exposure to increasing prenatal lead exposure (x-axis) and for high or low maternal depression score (dotted versus solid lines). High adjusted prenatal depression scores increased the probability that a child demonstrated a difficult temperament. When tibia lead concentration *and* adjusted prenatal depression score were low (25^th^ percentile or lower), a child demonstrated an easy or intermediate temperament approximately 70 % of the time. As prenatal lead exposure increased, the probability of demonstrating an easy or intermediate temperament fell and the probability of demonstrating a difficult temperament rose. This effect was more pronounced when the adjusted prenatal depression risk score was high. Similar results were seen when maternal blood lead was used as a measure of prenatal lead exposure, although results were less significant. Prenatal mercury exposure did not alter the effect of prenatal maternal depression on temperament (results not shown).

## Discussion

While associations between prenatal exposure to metals or maternal depression and cognitive development have been widely reported [12, 13, 20, 44–47], the role of such exposures *in early life behavioral development* is less well-understood. Effects seen in behavior early in life can help to parse the contribution of prenatal vs later life exposures, since exposures through the life-course are often correlated. Also, early life behavioral changes that result from environmental exposures may serve as intermediates for later life behavioral phenotypes, helping us to understand the complex interrelationships among exposure and behavioral development. Environmental health research focused on intelligence has generated a great appreciation for the impact of perinatal environmental exposures on ultimate IQ and intellectual achievement. However, learning and intellectual performance are complex processes that clearly interact with behavioral traits to produce psychological health. The importance of behavioral traits to overall child development is becoming increasingly apparent [73]. Cognition and cognitive performance is dependent on many behavioral traits such as attention and impulsivity that integrate with intellectual processes such as memory and mathematical ability in everyday life. To better understand central nervous system toxicity, the role of toxins in altering behavioral development and cognitive traits should be considered equally important. Prenatal and childhood exposure to maternal depression [74–76], lead [17, 77–79], and mercury [18, 22, 80] have been associated with behavior problems from middle childhood through adolescence. Measurable early life behaviors, such as temperament, predict psychiatric traits later in life including externalizing and internalizing disorders in school age children and ADHD [81, 82]. If early life temperament is predictive of later life psychiatric traits, then our results suggest that cumulative lead exposure and maternal depression may set these trajectories as early as age two. If we are to develop effective treatments or prevention measures for behavioral disorders, we need to both better understand the role of exposure timing and develop methods for early detection of deficits so that developmental plasticity can be leveraged to mitigate the neurotoxic effects of chemical and non-chemical exposures. Evaluation of temperament may provide this intermediate outcome as both sensitive to environmental exposures and measurable at a point in development when effective interventions can be initiated to reduce long-term morbidity.

Our data demonstrate a relationship between cumulative prenatal lead exposure and the likelihood of demonstrating a difficult temperament. Additionally, those mothers in our cohort with highest scores on prenatal depression screening had toddlers most likely to demonstrate difficult temperament traits, even after adjusting for maternal depression at the time of temperament assessment. As shown in Fig. 3, the association of cumulative prenatal lead exposure with difficult temperament was most pronounced among toddlers whose mothers had the highest adjusted prenatal depression scores. This indicates that both prenatal lead exposure and exposure to prenatal maternal depression impact the developing temperament of the child. The inclusion in our models of the mother’s prenatal depression score adjusted for the 24-month post-partum depression risk scores allows us to differentiate an association between prenatal depression risk and temperament from the known association between concurrent maternal depression and maternal report of difficult temperament. Prenatal mercury exposure and SES did not modify the effect of prenatal depression on infant temperament in our analyses.

The impact of *in utero* exposure to maternal depression and/or environmental chemicals on temperament and the interplay of multiple co-exposures on the establishment of temperament have not been evaluated previously. Our data suggest that cumulative lead exposure rather than a single second trimester lead measurement best predicts the lead-temperament association and that the joint impact of lead and prenatal depression is most predictive of difficult temperament. Tibia bone lead level, which has a half-life of 12 years or more, reflects cumulative lead exposure over the mother’s life-time [46, 83]. Previous studies have demonstrated that bone lead is a better predictor of infant development than blood lead, perhaps due to its longer half-life [48, 84]. However, bone lead cannot distinguish whether there is a specific window of sensitivity for lead exposure. Understanding the role of exposure timing could allow us to develop early intervention strategies.

As the PROGRESS cohort ages, we are measuring other neurobehavioral domains, such as attention, impulsivity, spatial memory, motivation, time estimation and internalizing/externalizing behaviors to further assess the impact of exposure to metals and maternal depression on child behavioral development. We will also address the role of temperament as a predictor of these neurobehavioral phenotypes.

Our study has a number of strengths. As a large, prospective birth cohort study focused on perinatal environmental exposures, both social and chemical environmental factors were assessed prospectively and longitudinally multiple times through pregnancy and early childhood. The EPDS, the BSID and the TTS are validated research measures that were administered by trained study staff, all of whom had masters or doctoral degrees in child psychology. Our statistical approach using LPA reduced the dimensionality of the multiple outcome subscales into a single 3 level index that clearly identified distinct phenotypes similar to those described in prior studies of infant temperament. The relationship of these three phenotypes with BSID performance further allowed us to validate our LPA profiles. Collecting multiple measures of lead exposure allowed us to distinguish between effects of blood lead level at a specific time point as well as cumulative lead.

Our study also has some limitations. The study population is composed of families in urban Mexico, and results may not be fully generalizable to other populations in other settings. Lead exposure in our cohort is generally higher and SES lower than in the United States, but this allows us to test our hypotheses more cost-effectively. Maternal depression was estimated by the EPDS, which is a screening rather than a diagnostic tool, so some women who had positive screens may not have met diagnostic criteria for clinical depression. As is the case for most assessment tools of temperament in childhood, the TTS relies on subjective maternal report.

## Conclusions

Prenatal exposure to maternal depression is associated with more difficult temperament traits at age two. This association is potentiated by concurrent prenatal exposure to environmental lead.

## Abbreviations

BIC, Bayesian information criteria; BSID, Bayley Scales of Infant and Toddler Development, 3rd edition; CI, confidence interval; EPDS, Edinburgh Postnatal Depression Scale; IQR, interquartile range; K-XRF, K-shell X-ray fluorescence; LPA, latent profile analysis; OR, odds ratio; SD, standard deviation; SES, socioeconomic status; TTS, Toddler Temperament Scale
